# One single method to produce native and Tat-fused recombinant human α-synuclein in *Escherichia coli*

**DOI:** 10.1186/1472-6750-13-32

**Published:** 2013-04-04

**Authors:** Laura Caldinelli, Diego Albani, Loredano Pollegioni

**Affiliations:** 1 Dipartimento di Biotecnologie e Scienze della Vita, Università degli Studi dell’Insubria, via J.H. Dunant 3, Varese, Italy; 2 The Protein Factory, Centro Interuniversitario di Biotecnologie Proteiche, Politecnico di Milano, ICRM – CNR Milano, and Università degli Studi dell’Insubria, via Mancinelli 7, Milano, Italy; 3 Dipartimento di Neuroscienze, Unità di Genetica delle Malattie Neurodegenerative, IRCCS - Istituto di Ricerche Farmacologiche Mario Negri, via La Masa 19, Milano, Italy

**Keywords:** α-Synuclein, TAT-fusion protein, Recombinant proteins, Parkinson’s disease, Oxidative stress, Protein aggregation

## Abstract

**Background:**

Human α-synuclein is a small-sized, natively unfolded protein that in fibrillar form is the primary component of Lewy bodies, the pathological hallmark of Parkinson’s disease. Experimental evidence suggests that α-synuclein aggregation is the key event that triggers neurotoxicity although additional findings have proposed a protective role of α-synuclein against oxidative stress. One way to address the mechanism of this protective action is to evaluate α-synuclein-mediated protection by delivering this protein inside cells using a chimeric protein fused with the Tat-transduction domain of HIV Tat, named TAT-α-synuclein.

**Results:**

A reliable protocol was designed to efficiently express and purify two different forms of human α-synuclein. The synthetic cDNAs encoding for the native α-synuclein and the fusion protein with the transduction domain of Tat protein from HIV were overexpressed in a BL21(DE3) *E. coli* strain as His-tagged proteins. The recombinant proteins largely localized (≥ 85%) to the periplasmic space. By using a quick purification protocol, based on recovery of periplasmic space content and metal-chelating chromatography, the recombinant α-synuclein protein forms could be purified in a single step to ≥ 95% purity. Both α-synuclein recombinant proteins form fibrils and the TAT-α-synuclein is also cytotoxic in the micromolar concentration range.

**Conclusions:**

To further characterize the molecular mechanisms of α-synuclein neurotoxicity both *in vitro* and *in vivo* and to evaluate the relevance of extracellular α-synuclein for the pathogenesis and progression of Parkinson’s disease, a suitable method to produce different high-quality forms of this pathological protein is required. Our optimized expression and purification procedure offers an easier and faster means of producing different forms (i.e., both the native and the TAT-fusion form) of soluble recombinant α-synuclein than previously described procedures.

## Background

Human α-synuclein (AS) is a 140-residue, natively unfolded protein that in fibrillar form is the primary component of Lewy bodies, the pathological hallmark of Parkinson’s disease (PD) [1]. PD is the second most common neurodegenerative disease resulting from the loss of dopaminergic neurons in the brain: it affects ≈ 2% of the population over the age of 65.

A structural and physiological understanding of AS fibrils at the molecular level is critical for finding cures for PD. Some evidence suggests that AS aggregation is the key event that triggers AS-mediated neurotoxicity [2,3]. However, other experimental data proposed a protective role of AS against oxidative stress (a major feature of PD) [4]. To investigate the exact mechanism underlying this protective action, as well as the role of AS pathogenetic mutations, different research groups developed *in vitro* models of oxidative stress based on the exposure of selected cells to stress (e.g., hydrogen peroxide, 6-hydroxydopamine, or serum deprivation) and evaluated AS-mediated protection by delivering AS inside cells using the fusion protein TAT-AS (for details, see below) [5]. These studies established that nanomolar amounts of TAT-AS protected against stress and increased Hsp70 protein levels, whereas micromolar amounts of the protein were intrinsically toxic to cells and decreased Hsp70 at the protein level.

Recombinant AS has been produced in *Escherichia coli* since 1994 [6] by different methods which employ whole-cells extract as the protein source [7], followed by ammonium sulfate or acid precipitation and successive chromatographic steps [8,9]. Alternative strategies are based on the design of fusion proteins - for example, with the glutathione S-transferase or the chitin binding domain - and the use of specific enzymes for releasing the fused AS [10,11]. Most recently, AS has been overexpressed within *E. coli* periplasm and from this compartment it was recovered in the native form by only two purification steps [12]*.* AS was also produced as recombinant protein fused with transduction domain of Tat protein from HIV. In fact, it was demonstrated that heterologous proteins chemically crosslinked to a domain of Tat protein from HIV were able to transduce into cells [13]: accordingly, this method become widely used because full-length proteins can be rapidly introduced into primary and immortalized cells. The fusion proteins can be directly added to cell culture [14,15] or injected *in vivo* into mice [16]. Protein transduction occurs in a concentration-dependent manner, requires at least one hour to achieve maximum intracellular concentrations with nearly equal intracellular concentrations among all cells in the transduced population and the uptake does not depend on the cell type used [17,18]. The technology requires the synthesis of a fusion protein, linking the transduction domain (the minimal transduction domain is represented by residues 47–57 of HIV Tat, named TAT) [16] to the molecule of interest by using a bacterial expression vector, followed by the purification of this fusion protein under either soluble or denaturing conditions. For a review, see [19,20]. TAT-AS was previously produced by expressing pTAT/pTAT-HA in a BL21(DE3)pLysS *E. coli* strain [5]. It was purified from a washed bacterial pellet suspended in 8 M urea followed by metal affinity chromatography on a Ni-NTA chelating column because of the hexahistidine tag added during the cloning procedure, following the guidelines reported in [17].

To provide a tool to further characterize the molecular mechanisms of AS neurotoxicity both *in vitro* and *in vivo* and to evaluate the relevance for PD pathogenesis and progression of extracellular AS, we focused on setting up a fast, simple, and inexpensive method of producing high-quality AS, both the native and the TAT-fusion AS form.

## Results and discussion

### Expression of recombinant AS in *E. coli*

Synthetic cDNA encoding for native or TAT-AS (GenBank code KC609369 and KC609370, respectively) was subcloned into pET11a plasmid, giving a construct encoding for a protein that was 148- and 170-amino acids in length (AS or TAT-AS, of 15.5 or 17.8 kDa), both containing an N-terminal His-tag.

In order to optimize AS expression, several experimental parameters were modified at the flask scale using LB medium: IPTG concentration (0, 0.1, or 0.5 mM), growth phase at induction (OD_600nm_ = 0.4 or 0.8), and time of cell harvest after adding IPTG (up to 24 hours). Varying incubation times after adding IPTG significantly affects expression of both AS recombinant forms obtaining maximum protein production after five hours interval; no AS production is observed before IPTG is added (Additional file 1: Figure S1). Comparatively slightly higher (≈ 1.3-fold) amounts of AS are produced if expression is induced at an OD_600nm_ ≈ 0.4 than at the later exponential phase of growth. No increase in AS expression level was observed using a richer medium (i.e. YT and SB media) or decreasing the temperature of growth after IPTG addition. The latter observation agrees with previous studies that reported the highest production of recombinant AS at 37°C [12,21]: the rationale for the lack of temperature dependence is probably an effect of the small size of the investigated protein [22]. Taken together, Western blot analysis shows that the highest production of both native AS and TAT-AS in *E. coli* (≈ 35 and 22 mg per liter of fermentation broth, respectively) is observed by growing the cells in LB medium to an OD_600nm_ = 0.4, adding 0.1 mM IPTG, and growing for another 5 hours at 37°C. The best conditions set-up was then scaled up to a 2-L flask level containing 500 mL of LB broth.

The overexpressed native and TAT-AS are largely (≥ 85%) produced as soluble proteins, as demonstrated by SDS-PAGE of soluble and insoluble cell content following sonication (Additional file 1: Figure S2A). Indeed, recombinant AS is largely targeted to the periplasmic space where it represents ≈ 70% of the whole protein content (Additional file 1: Figure S2B). In fact, although no signal sequence is apparent for translocation into the periplasmic space, the C-terminal 99-to-140 portion of the protein plays a signal-like role, cooperating with the central 61-to-95 protein region [23].

### Purification and characterization of recombinant AS

**Figure 1:**
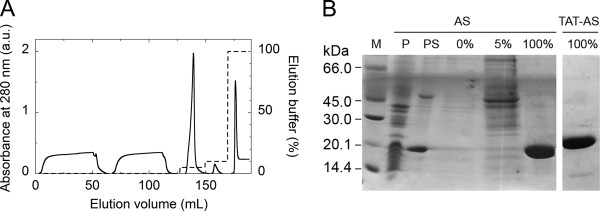
**Representative purification of recombinant native AS on HiTrap chelating column (5 mL). A**) An amount of periplasmic sample corresponding to 2 L of fermentation broth was loaded and eluted in 20 mM sodium phosphate, pH 7.4, 1 M NaCl. Following two washing steps at 5 and 10% of elution buffer to remove proteins aspecifically bound to the column, AS was eluted with 500 mM imidazole in 20 mM sodium phosphate, pH 7.4 (elution buffer). **B**) SDS-PAGE analysis of fractions eluted from HiTrap chelating column. P and PS: cell pellet after osmotic shock and periplasmic space content (both corresponding to 0.2 mL of fermentation broth); 0%, 5% and 100%: fractions eluted at different percentage of elution buffer; M: molecular mass standard proteins (GE Healthcare). TAT-AS: purified TAT-AS recombinant protein eluted at 100% of elution buffer.

Starting from 2 L of fermentation broth, native AS was purified by employing only two steps: osmotic shock to recover the periplasmic space content followed by chromatography on a HiTrap chelating column. As shown in Figure 1A, following preliminary washing steps at 5-10% of elution buffer (i.e., 25–50 mM imidazole), the recombinant AS is eluted at 100% of elution buffer as a single, narrow peak. An overall yield of ≈ 16 mg native AS/L of fermentation broth is achieved with a > 95% purity of the final preparation as judged by SDS–PAGE (Figure 1B). Interestingly, TAT-AS could also be purified to a high degree under the same experimental conditions (> 95%, see Figure 1B) and gave a yield of up to 12 mg/L of fermentation broth (Table 1).

**Table 1:** **Purification of native AS and TAT-AS from *E. coli *starting from 1 L culture (≈ 2.5 g cell paste)**

**Purification step**	**Total protein (mg)**	**AS (mg)**	**AS purity **^**a **^**(%)**	**Yield **^**b **^**(%)**
Whole cell extract ^c^	210 [185] ^d^	35 [22.2]	16.7 [12.0]	100 [100]
Osmotic shock	40 [29] ^d^	30 [20]	75 [69]	85 [90.1]
HiTrap chromatography	16 [11.8] ^e^	16 [11.8]	> 95 [> 95]	53 [59]

The value obtained for the purification of TAT-AS is shown in brackets.

^a^ The purity of AS at each step of purification was estimated by SDS–PAGE.

^b^ Ratio of the amount of AS obtained at each step to the total amount of AS estimated in the whole cell extract.

^c^ Supernatant of the cell lysate by sonication.

^d^ Determined by Bradford assay with bovine serum albumin as a standard.

^e^ Determined spectrophotometrically.

**Figure 2:**
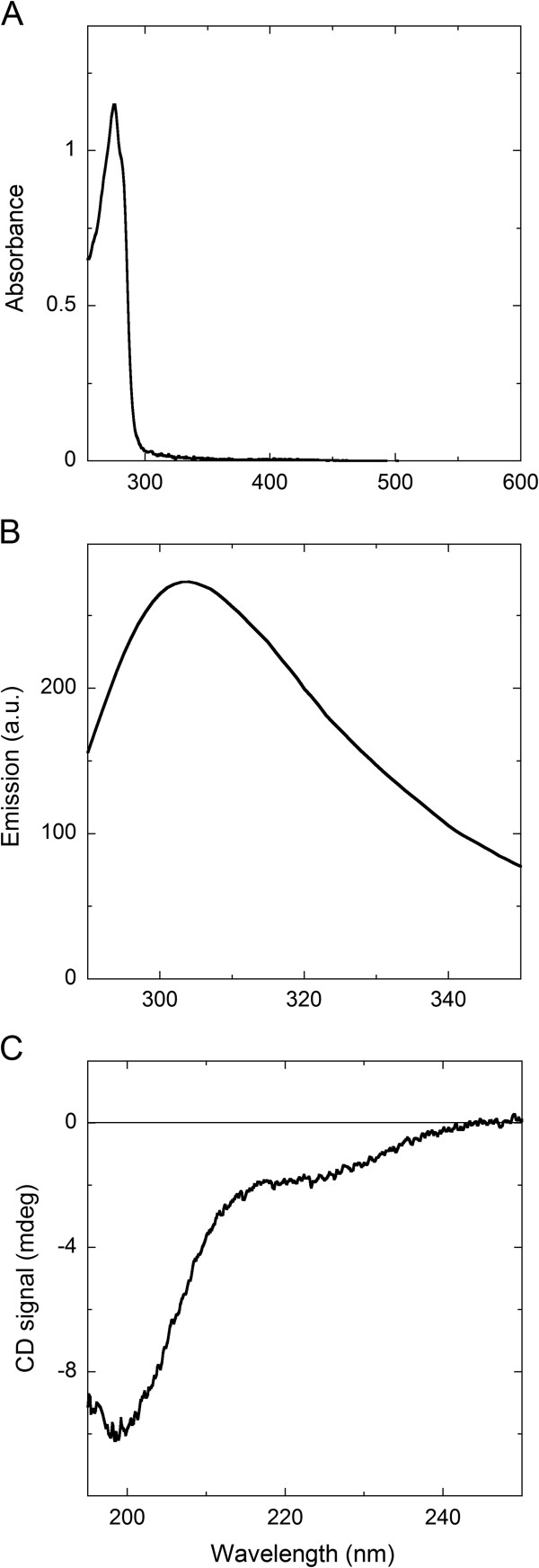
**Spectral properties of recombinant native AS.** In 20 mM potassium phosphate buffer, pH 7.4, 150 mM sodium chloride, after eliminating endotoxins. **A**) UV–vis Absorbance spectrum of AS, 3.2 mg/mL, 15°C. **B**) Protein fluorescence of AS; 0.1 mg/mL, excitation at 280 nm. **C**) Far-UV circular dichroism spectrum of AS; 0.1 mg/mL.

The endotoxins (lipopolysaccharides) were completely eliminated from the final protein preparation by treating it with Triton X-114 (see Methods section) as assessed by the E-TOXATE test. The absence of the detergent in the final AS sample was apparent from the absorbance spectrum, which showed a maximum at 276 nm (Figure 2A).

The intrinsic fluorescence spectrum of purified recombinant AS showed a maximum emission at 305 nm (Figure 2B), as reported previously [24]. The circular dichroism spectrum of both purified AS forms presented a strong negative peak at 202 nm and a small shoulder at around 222 nm (Figure 2C), indicating a random coil structure possibly with short, marginally stable α-helices; see also [25].

**Figure 3:**
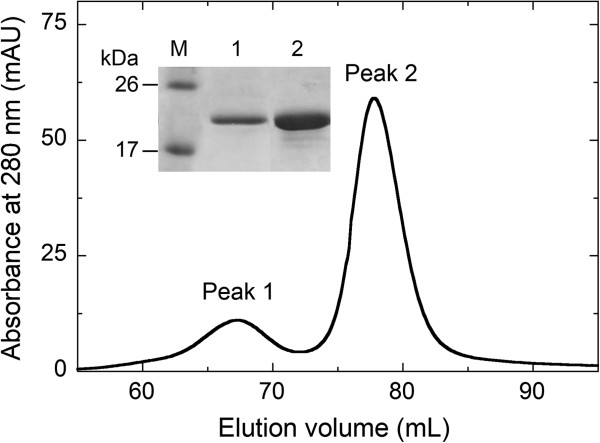
**Gel permeation analysis of recombinant TAT-AS.** Peak 1 corresponds to an apparent molecular mass of 130 ± 8 kDa and peak 2 corresponds to an apparent molecular mass of 61.4 ± 5.4 kDa; both peaks gave a single band at 18 kDa in SDS-PAGE analysis (inset). Conditions: HiLoad 16/60 Superdex 200 (GE Healthcare) column; 20 mM potassium phosphate buffer, pH 7.4, 150 mM sodium chloride as elution buffer.

AS is known to be a monomer using nondissociating PAGE [8] but it elutes in size-exclusion chromatography as a symmetric peak at a position corresponding to a larger apparent molecular mass [26]. Gel-permeation chromatography of purified recombinant native AS and TAT-AS gave a main peak at 61.5 ± 5.4 kDa (expressed as mean ± SD, n = 4, Figure 3): the abnormal behavior of AS in size-exclusion chromatography has been ascribed to its natively unfolded conformation in physiological buffer [8]. Indeed, an additional minor peak (≈ 14% of the main peak) corresponding to an apparent molecular mass of 130 ± 8 kDa was also apparent: both peaks gave a single band at 18 kDa in SDS-PAGE analysis (inset of Figure 3). The peak at lower elution volume was previously demonstrated to correspond to a dimer of AS due to disulfide bond formation between cysteines at position 136 under non-reducing conditions [27]. Misincorporation of Cys instead of Tyr at position 136 was reported to happen in AS with a 20-25% frequency: because of the reducing environment of the final cellular destination, dimeric AS should convert into the monomeric form thus not affecting the cell assay.

### Atomic force microscopy and functional evaluation of purified AS

**Figure 4:**
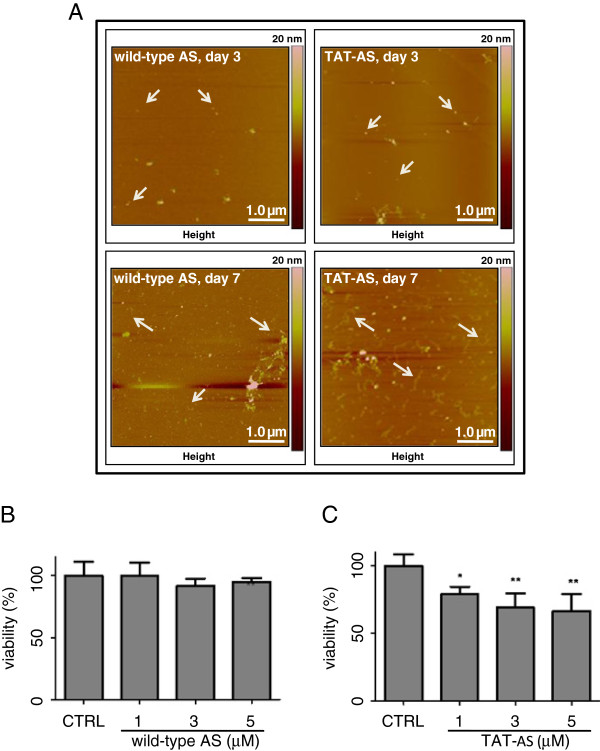
**Functional validation of purified AS. A**) Atomic force microscopy (AFM) of native AS and TAT-AS. AS was incubated as described in Methods to trigger aggregation and fibrillogenesis. At day 3, arrows highlight oligomeric species, while at day 7 they point to longer fibrils. **B**-**C**) Cell toxicity assay of purified AS. SHSY-5Y cells were exposed to increasing amounts of AS native (**B**) or TAT-AS (**C**) for 24 hours; then cell viability was measured by MTT colorimetric test. Each experimental condition was conducted in quadruplicate. CTRL: control; *p<0.05; **p<0.01, one-way ANOVA and Tukey’s *post-hoc* test.

AS is natively unfolded and prone to aggregation. To evaluate the fibrillogenic propensity of purified AS, we incubated AS (both native and TAT) for increasing time intervals (2 up to 7 days) at 37°C with sporadic mild agitation. Aliquots of the preparations were then analyzed by atomic force microscopy (AFM). Starting from day 2 and up to day 3, we observed AS aggregation with clearly detectable oligomers, while fibrils were formed after longer incubation times. Qualitatively, the TAT-fused AS form seems more fibrillogenic than the native counterpart (Figure 4A).

To functionally assess the purified AS, we performed a toxicity assay on SHSY-5Y cells. Cells were incubated with increasing amounts of AS (both native and TAT) for 24 hours; then viability was measured by 3-(4,5-dimethylthiazol-2-yl)-2,5-diphenyltetrazolium bromide (MTS) colorimetric assay (Figure 4B-C). We confirmed the toxicity of TAT-AS whereas the native form had no toxic effect in this kind of assay, as previously reported [5]. The basic toxicity of TAT-AS might be a starting point for experiments aimed at unraveling the relation between AS and other kind of cellular stressors, for instance oxidative stress, proteasome impairment or autophagy, all features potentially linked to PD etiopathogenesis [28-31].

## Conclusions

Mammalian cells can be manipulated by transfecting expression vectors, microinjection, or diffusion of peptidyl mimetics: these approaches have been rather successful but are not easily regulated and can be laborious. One approach to circumvent these problems is to use HIV Tat-mediated protein transduction as initially described by [32,33]. We report on the expression and purification of AS, a protein implicated in PD, which exists as a natively unfolded protein in physiological buffer. Here, a new protocol for preparing recombinant native AS and TAT-AS has been developed that involves two steps only: (1) osmotic shock for release of AS-containing periplasm fraction and (2) HiTrap chelating chromatography for further purification of AS. Previous methods used to produce recombinant native AS in *E. coli* required more steps, comprising at least one ammonium sulphate precipitation and two chromatographic separations [12,21,27]. Noteworthy, and differently from previously reported methods [17], TAT-AS was largely produced as soluble protein thus avoiding the need for a resolubilization step. This result is due to a controlled rate of protein synthesis – because of the low IPTG concentration added at the exponential phase of growth – and to the efficient targeting of TAT-AS to the periplasmic space. The latter is not an artifact of massive overproduction or of the experimental method of osmotic shock: AS in the periplasm of *E. coli* exists stably in (largely) disordered monomeric form [23]. The secondary, tertiary, and (apparent) quaternary structures of both native and TAT-fusion AS forms correspond to those of known protein. Indeed, both recombinant AS proteins form fibrils and TAT-AS only shows cytotoxic effects on SHSY-5Y cells.

This new protocol is a convenient, economical, and rapid procedure for preparing different forms of AS, which favourably compares to methods currently being used. About 12–16 mg AS (native and TAT-fusion protein) with 95% purity can be regularly prepared from a 1-L culture in 1 day. AS is also commercially available (at a cost > 600 $/mg protein) but its variant forms (such as the TAT-AS required for advanced studies) are not.

In conclusion, the recombinant AS forms produced here represent a useful tool for *in vitro* or *in vivo* experiments where a tight control of AS concentration and intracellular or extracellular localization is mandatory. Moreover, the capability of native AS and TAT-AS to aggregate and form oligomers or fibrils, though with different toxic effect on our cell model, is of pivotal importance in order to discriminate the real toxic intermediate and the relevance of cellular localization in the dynamics of AS aggregation, toxicity and cell-to-cell propagation, a still debated question in the field of PD pathophysiology.

## Methods

### Design and cloning of AS cDNAs

Two synthetic cDNAs for AS (one encoding for the native protein and the second containing the additional sequence encoding for the Tat transduction domain, GenBank code KC609369 and KC609370, respectively) were designed by *in silico* back translation of the amino acid sequence reported in the data bank (GenBank accession number NM_001146055.1, *α-syn* gene) and optimizing the codon usage for *E. coli* expression. In detail, all AAG codons (encoding for Lys) were converted into AAA; all CTT, CTC, and TTG codons (encoding for Leu) were converted into CTG; one GGA codon (encoding for Gly) was converted into GGT; and one GTG codon (encoding for Val) was converted into GTT. In order to facilitate the subcloning into pET11a plasmid (carrying the resistance to ampicillin, Novagen), *NdeI* (CATATG) and *BamHI* (GGATCC) sites were added at the 5’- and 3’-ends of the cDNA, respectively, and a sequence encoding for six additional histidines was added to the 5’-terminal end of both AS protein forms. The *α-syn* cDNAs were inserted in the pET11a vector using *NdeI* and *BamHI* sites, giving 6.088- and 6.154-kb constructs (pET11-AS and pET11-TAT-AS, respectively).

### Strains and growth conditions

For protein expression, both recombinant plasmids were transferred to the *E. coli* host BL21(DE3) strain: starter cultures were prepared from a single recombinant *E. coli* clone in LB medium containing ampicillin (100 μg/mL final concentration). Expression trials were conducted using baffled (500 mL) Erlenmeyer flasks containing 100 mL of LB, SB or YT medium that was inoculated with the starter culture (initial OD_600nm_ = 0.05); cells were grown at 37°C and shaken (180 rpm) until the protein expression was induced by adding IPTG. Crude extracts were prepared by sonication; see [34] for details. The insoluble fraction of the lysate was removed by centrifugation at 39,000 *g* for 1 hour at 4°C. Expression level of AS was assessed by SDS-PAGE and Western blot analysis; see below.

For protein purification, periplasmic space content was recovered by adding 50 mL of osmotic shock buffer (30 mM TrisHCl, pH 7.2, 40% saccharose, 2 mM EDTA) to 2.5 g of cell paste (from 1 L of fermentation broth) and incubating for 10 min at room temperature; cells were collected by centrifugation at 17,200 *g* for 20 min and the pellet was quickly dissolved in 45 mL of cold 1 mM MgCl_2_. The protein content of the periplasmic space was released and after 3 min at 4°C collected by centrifugation at 17,200 *g* for 20 min. To the soluble extract 5 mL of 200 mM sodium phosphate was added, pH 7.4, containing 20 μg/mL leupeptin, 7 μg/mL pepstatin, and 1.9 μg/mL PMSF.

### Purification of the recombinant AS

Both recombinant AS forms containing an N-terminal His-tag were purified from soluble extracts using Ni^2+^-affinity chromatography (HiTrap Chelating HP columns, GE Healthcare) equilibrated with 20 mM sodium phosphate, pH 7.4, 1 M NaCl. The bound protein was eluted with 500 mM imidazole in 20 mM sodium phosphate, pH 7.4. Imidazole was then removed by overnight dialysis at 4°C against 20 mM sodium phosphate buffer, pH 7.4, 150 mM sodium chloride.

The amount of purified AS was determined using the extinction coefficient at 280 nm of 5960 M^-1^ cm^-1^ for native AS and of 7450 M^-1^ cm^-1^ for TAT-AS. The amount and purity degree of the final AS preparations were also estimated by SDS-PAGE and densitometric analysis using the software Quantity One (Biorad). Recombinant AS was analyzed by Western blot using anti-His-tag mouse monoclonal antibodies (His-probe, Santa Cruz Biotechnology) and goat anti-mouse IgG HRP-conjugated antibodies (Jackson ImmunoResearch) [34,35]. Recognition was confirmed by employing a chemiluminescent test (ECL Plus Western Blotting Detection System, GE Healthcare), using His-tagged D-amino acid oxidase as positive control [36].

Safety precautions during production and managing of TAT-AS were as detailed in [17].

Endotoxins were removed from the final AS preparation according to the procedure suggested by [37]. Briefly, 1% Triton X-114 was added to the protein sample, which was incubated at 4°C for 30 min and then at 37°C for 10 min, and finally centrifuged at 16,000 *g* for 15 min. The aqueous fraction was recovered and the incubation at 37°C, the centrifugation step, and recovery of aqueous fraction were repeated until Triton X-114 was completely eliminated. The removal of endotoxins was assessed by the E-TOXATE test (Sigma-Aldrich).

### Biochemical characterization of recombinant AS

UV-Visible absorption spectra were recorded with a Jasco V-560 spectrophotometer (Jasco Co., Cremello Italy). Circular dichroism spectra were recorded on a J-810 Jasco spectropolarimeter and analyzed by means of Jasco software; cell pathlength was 0.1 cm for measurements in the 190- to 250-nm region (0.1 mg protein/mL) [38]. All measurements were carried out at 15°C in 20 mM sodium phosphate, pH 7.4, 150 mM sodium chloride. Baseline was corrected to account for the buffer content. Circular dichroism spectra were analyzed by K2d software [39].

Size-exclusion chromatography was performed at room temperature on a Superdex 200 or HiLoad Superdex 200 (GE Healthcare) column by means of an Äkta chromatographic system (Amersham Pharmacia Biotech), using 20 mM potassium phosphate, pH 7.4, 150 mM sodium chloride, as elution buffer. The column was calibrated with suitable standard proteins.

### Atomic force microscopy

AS 130 μM was incubated at 37°C for increasing time intervals (2, 3 and 7 days). Each sample was diluted to 50 μM with 10 mM phosphate-buffered saline, pH 7.4, and incubated for 0.5 min on a freshly cleaved Muscovite mica disk. After the incubation period, the disk was washed with H_2_O and dried under a gentle stream of nitrogen. The sample was mounted onto a Multimode AFM with a NanoScope V system operating in Tapping Mode using standard phosphorus-doped silicon probes (T: 3.5–4.5 lm, L: 115–135 lm, W: 30–40 lm, K: 20–80 N/m) (Veeco Instruments, Plainview, NY, USA).

### AS cell toxicity assay

SHSY-5Y cells were cultured at 37°C, 5% CO_2_ in D-MEM supplemented with 10% fetal bovine serum (FBS), 2 mM L-glutamine, 100 IU/mL penicillin, and 100 μg/mL streptomycin (Invitrogen, Carlsbad, CA, USA). Then, 3 x 10^4^ cells were seeded in a 96-well plate and incubated overnight. The next day, the medium was changed and the toxicity of AS was assessed by adding increasing amounts at the μM scale of the purified protein (both native and TAT) for 24 hours. Then, cell viability was measured by a MTT-based assay according to the manufacturer’s instructions (Promega Corp., Madison, WI, USA).

## Abbreviations

AFM: Atomic force microscopy; AS: α-synuclein; MTT: 3-(4,5-dimethylthiazol-2-yl)-2,5-diphenyltetrazolium bromide; PD: Parkinson’s disease; TAT-AS: chimeric α-synuclein protein fused with the Tat-transduction domain of HIV Tat.

## Competing interests

The authors declare that they have no competing interests.

## Authors’ contributions

LC carried out the cloning, expression, and purification of α-synuclein forms and drafted the manuscript. DA planned the atomic force microscopy and cell toxicity assays and participated in preparing the manuscript. LP supervised the study, planned the experiments, and wrote the manuscript. All authors read and approved the final manuscript.

## Supplementary Material

Additional file 1: Figure S1 Analysis of expression of TAT-AS in BL21(DE3) *E. coli* cells by means of Western blot. Protein expression was induced at an OD_600nm_ = 0.4, adding 0.1 mM IPTG; the cells were then grown for the time indicated at 37 °C. **Figure S2.** Subcellular distribution of AS forms overexpressed on *E. coli*. A) SDS-PAGE analysis of protein content in whole cell fraction (C), crude extract (following sonication and centrifugation, CE) and pellet (insoluble fraction, P) of BL21(DE3) *E. coli* cells expressing native AS (left) or TAT-AS (right). B) Subcellular distribution of overexpressed AS forms: (C) whole cell fraction, (PS) periplasmic space. Click here for file
