# Supplementary material for: One single method to produce native and Tat-fused recombinant human α-synuclein in Escherichia coli
Source: BMC Biotechnol. 2013 Apr 4;13:32. doi: 10.1186/1472-6750-13-32 (PMC3621789; doi:10.1186/1472-6750-13-32)
Supplement: Additional file 1: Figure S1 — Analysis of expression of TAT-AS in BL21(DE3) E. coli cells by means of Western blot. Protein expression was induced at an OD600nm = 0.4, adding 0.1 mM IPTG; the cells were then grown for the time indicated at 37 °C. Figure S2. Subcellular distribution of AS forms overexpressed on E. coli. A) SDS-PAGE analysis of protein content in whole cell fraction (C), crude extract (following sonication and centrifugation, CE) and pellet (insoluble fraction, P) of BL21(DE3) E. coli cells expressing native AS (left) or TAT-AS (right). B) Subcellular distribution of overexpressed AS forms: (C) whole cell fraction, (PS) periplasmic space. [file 1472-6750-13-32-S1.pdf]

Additional File 1

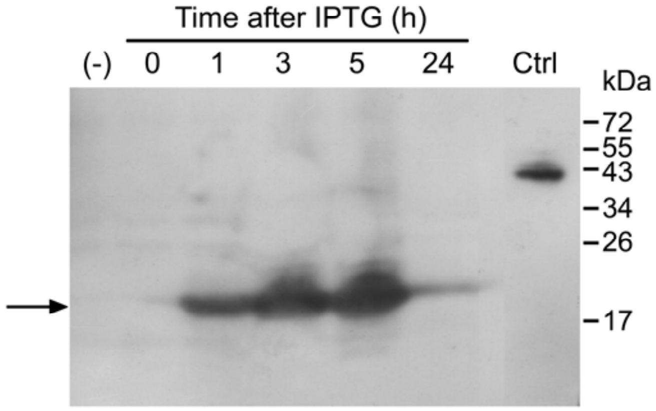

Figure S1. Analysis of expression of TAT-AS in BL21(DE3) *E. coli* cells by means of Western blot. Protein expression was induced at an OD_{600nm} = 0.4, adding 0.1 mM IPTG; the cells were then grown for the time indicated at 37 °C. (-): Total cell extract of untransfected *E. coli* cells (negative control). Arrow indicates the position of the mature protein. A sample corresponding to 0.2 mL of culture was loaded in each lane. Ctrl: 0.5 µg of His-tagged recombinant D-amino acid oxidase.

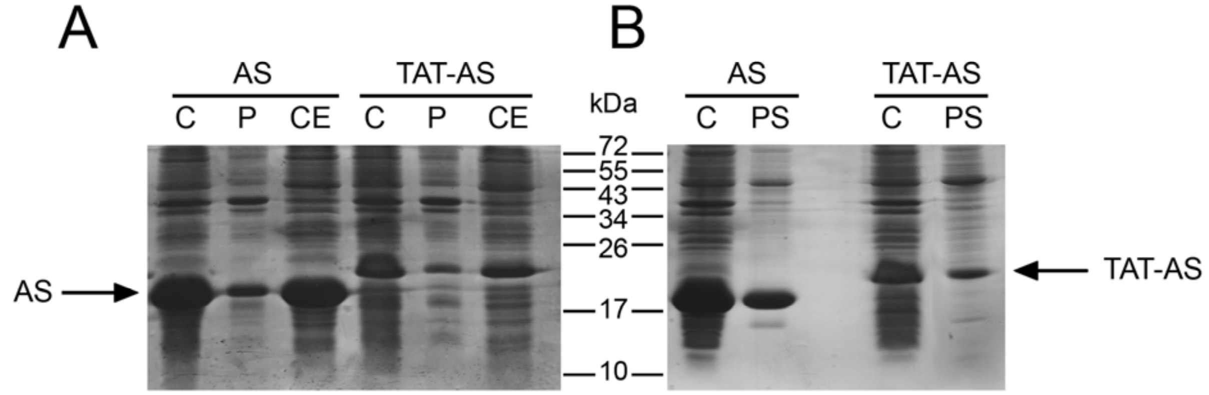

Figure S2. Subcellular distribution of AS forms overexpressed on *E. coli*. A) SDS-PAGE analysis of protein content in whole cell fraction (C), crude extract (following sonication and centrifugation, CE) and pellet (insoluble fraction, P) of BL21(DE3) *E. coli* cells expressing native AS (left) or TAT-AS (right). B) Subcellular distribution of overexpressed AS forms: (C) whole cell fraction, (PS) periplasmic space. Arrows indicate the position of the mature proteins. A sample corresponding to 0.2 mL of culture was loaded in each lane.
